# Associations of Biotic and Abiotic Factors with Phylogenetic Community Structure Across Temperate Forests in South Korea

**DOI:** 10.3390/biology15030268

**Published:** 2026-02-02

**Authors:** Chang-Bae Lee

**Affiliations:** 1 Department of Forest Resources, Kookmin University, 77 Jeongneung Rd., Seongbukgu, Seoul 02707, Republic of Korea; kecolee@kookmin.ac.kr; 2 Department of Climate Technology Convergence, Kookmin University, 77 Jeongneung Rd., Seongbukgu, Seoul 02707, Republic of Korea; 3 Forest Carbon Graduate School, Kookmin University, 77 Jeongneung Rd., Seongbukgu, Seoul 02707, Republic of Korea

**Keywords:** community weighted mean traits, phylogenetic community structure, species richness, deterministic and random processes, temperate forests

## Abstract

Understanding how environmental conditions, functional composition, and species richness jointly relate to phylogenetic community structure is important for sustainable forest management under environmental change. Using 2858 plots from the 7th National Forest Inventory of South Korea, phylogenetic community structure as the standardized effect size of mean pairwise phylogenetic distance (SES.MPD) was quantified for broadleaved, conifer, and mixed stands. Associations between SES.MPD and abiotic factors such as elevation, mean annual precipitation, stand age, as well as biotic factors such as species richness and community-weighted means of specific leaf area and maximum height were assessed using multi-model inference and piecewise structural equation models (pSEM). Across stand types, SES.MPD values in most plots were not significantly different from the null-model baseline under the chosen randomization procedure, indicating weak net departures from null-model-relative phylogenetic dispersion at the national scale; meanwhile, mean SES.MPD tended to be slightly negative in broadleaved stands and positive in conifer and mixed stands. In multi-model inference analysis, the strongest associations with SES.MPD differed among stand types: trait composition—especially community-weighted specific leaf area—showed the strongest association in total stands and broadleaved stands, whereas species richness was the dominant correlate in mixed stands and precipitation showed the strongest association in conifer stands. The pSEM revealed that elevation, precipitation, and stand age were linked to SES.MPD largely through indirect pathways via trait composition and species richness, consistent with trait- and richness-mediated environmental filtering. These results highlight stand-type-specific linkages among environment, traits, richness, and phylogenetic structure and support trait- and phylogeny-informed forest management and restoration to enhance resilience under ongoing environmental change.

## Simple Summary

Forests contain many different tree species that are linked to each other through a long evolutionary history. Which species grow together in a given stand depends not only on the climate and soil, but also on chance events such as where seeds arrive and how past disturbances have reshaped the forest. In this study, a nationwide forest inventory and phylogenetic trees of all recorded woody species were used to examine how Korean temperate forests are assembled and which factors matter most in different forest stand types. More than two thousand forest plots across broadleaved, conifer, and mixed stands were analyzed, incorporating both environmental conditions and key tree traits, such as specific leaf area and maximum tree height, and species richness. Overall, SES.MPD in most plots showed weak net deviation from the null-model baseline, while the main correlates of phylogenetic community structure differed among stand types: functional trait-related variables were most important in broadleaved and conifer stands, whereas species richness played the dominant role in mixed stands. These results provide a basis for designing forest management and restoration strategies that combine species with diverse traits and evolutionary histories, helping to build forests that are more resilient to future climate change, pests, and other disturbances.

## 1. Introduction

Understanding which species occur in an ecological community and in what combinations they co-occur has long been a central question in ecology [1,2]. In recent decades, research has increasingly moved beyond simple analyses of species richness and composition to use phylogenetic community structure to infer the mechanisms underlying community assembly [1,2,3]. Because phylogenetic information encapsulates the shared evolutionary history of species and the trajectories of trait evolution, it provides a powerful framework for indirectly inferring various community assembly processes—such as environmental filtering, interspecific competition, and dispersal limitation—from observed community patterns [1,4].

Community assembly is commonly described as the outcome of interacting deterministic and random (or stochastic) processes operating along a continuum whose relative importance varies with environmental conditions [5,6]. Deterministic processes such as environmental filtering, niche partitioning, and interspecific competition, favor species that match local abiotic conditions (e.g., climate, elevation, soils) and biotic interactions [6], whereas random processes including dispersal limitation, stochastic colonization and local extinction, and disturbance legacies can also shape which species arrive, persist, and spread particularly when species have similar fitness or when environments are heterogeneous [5,7]. Phylogenetic community structure can be used to indirectly summarize the net imprint of these processes when evaluated relative to a specified null model. Importantly, interpretations of phylogenetic clustering versus overdispersion are scale-dependent and null-model dependent, because the direction and magnitude of SES-based patterns can vary with the spatial extent of analysis and with assumptions about the species pool and randomization procedure. Accordingly, strong filtering can yield phylogenetic clustering when ecologically similar (and often closely related) species are repeatedly selected, whereas stronger limiting similarity or competitive exclusion may promote coexistence among more distantly related species, producing phylogenetic overdispersion [1,4]. In contrast, when opposing processes offset one another or when stochasticity and historical contingencies are prominent, phylogenetic structure may show weak or inconsistent departures from null expectations and may be only modestly explained by measured environmental or trait variables [3,8,9]

Analyzing phylogenetic community structure helps interpret community patterns beyond species counts by linking species relatedness to environmental gradients, functional strategies, and ecosystem functioning when key traits show phylogenetic signal [2,3,4,10,11,12]. Accordingly, integrating abiotic variables with biotic descriptors such as species richness and community-weighted mean (CWM) traits (e.g., specific leaf area and maximum height) can help interpret how environmental filtering, biotic interactions, and other processes jointly shape forest communities [3,13,14]. However, much of the existing literature has emphasized abiotic gradients (e.g., climate, elevation, soils) while less consistently incorporating biotic variables and stand development context (e.g., stand age, successional stage) into a unified analytical framework [3,8,9]. This imbalance can bias interpretation toward environmental filtering and makes it difficult to assess whether observed phylogenetic patterns primarily reflect trait-mediated filtering, competition and niche differentiation, or dispersal limitation. Consequently, there remains a need for integrative, stand-type-comparative analyses that jointly evaluate abiotic conditions, functional composition, species diversity, and stand development stage to better explain variation in phylogenetic structure across forest types [6,15,16,17]. In Korea, most prior phylogenetic community studies have largely been conducted at local extents or along specific gradients (e.g., elevation), whereas a nationwide, stand-type-comparative assessment that integrates abiotic gradients with trait composition, diversity, and stand development using NFI plots has been limited [12,17].

In this context, this study used 2858 plots of the 7th National Forest Inventory of South Korea to quantify phylogenetic community structure across broadleaved, conifer, and mixed temperate forests and to evaluate how abiotic factors (elevation, mean annual temperature and precipitation, and stand age) and biotic factors (species richness, CWM.SLA, and CWM.MH) are associated with the standardized effect size of mean phylogenetic distance (SES.MPD). Because SES.MPD describes phylogenetic dispersion relative to a specified null model—rather than directly identifying assembly mechanisms—the aim was to identify stand-type-specific correlates and linkage pathways instead of attributing patterns to a single process. Three research questions were addressed: (Q1) Do mean SES.MPD patterns differ among broadleaved, conifer, and mixed stands? (Q2) Are biotic attributes (species richness and trait composition) stronger correlates of SES.MPD than abiotic gradients at the national scale, and does this differ among stand types? and (Q3) Are associations between abiotic gradients and SES.MPD expressed primarily through indirect pathways mediated by trait composition and species richness? These questions were evaluated using three testable expectations: (H1) mean SES.MPD differs among stand types, reflecting contrasts in dominant lineages and community composition; (H2) biotic predictors—particularly trait composition and/or species richness—show stronger associations with SES.MPD than abiotic gradients in at least some stand types; and (H3) abiotic gradients are linked to SES.MPD partly through indirect, trait- and richness-mediated pathways. H2 is evaluated using model-averaged standardized coefficients (direct associations) from multi-model inference, whereas H3 evaluated using pSEM to examine indirect, trait- and richness-mediated pathways.

## 2. Materials and Methods

### 2.1. Study Area and Data

South Korea is located in the temperate zone, with a mean annual temperature of 7–15 °C and mean annual precipitation of approximately 1000–1900 mm, and about 63% of the national territory is covered by forests [18]. Forests with stand ages of 41–50 years account for about 40% of the total forest area, and coniferous, broadleaved, and mixed forests represent approximately 37%, 32%, and 26% of the forest area, respectively [19]. The main conifer tree species are *Pinus densiflora* and *Pinus thunbergii*, whereas broadleaved forests are dominated by oaks such as *Quercus mongolica*. Mixed forests generally represent stands where *P*. *densiflora* forests are undergoing succession toward *Q*. *mongolica* forests, and both species usually co-occur.

**Figure 1:**
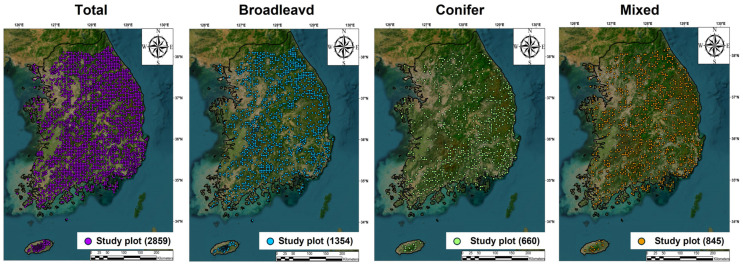
Location of National Forest Inventory (NFI) study plots (400 m^2^) across South Korea, classified into total, broadleaved, conifer, and mixed stands.

This study used data from the 7th National Forest Inventory (NFI) of Korea, conducted by the Korea Forest Service and the Korea Forestry Promotion Institute between 2016 and 2020, covering all forests in the country (Figure 1). In the NFI, the whole territory of South Korea is divided into a 4 km × 4 km grid, and at each grid vertex a cluster comprising four circular plots (each with a radius of 11.3 m and an area of 400 m^2^) is established. Since the 5th NFI (2005–2010), vegetation surveys have been repeatedly conducted every five years in the same clusters [19]. In each cluster, one central plot is located at the grid vertex, and three additional plots are placed 50 m away from the central plot at bearings of 0°, 120°, and 240°. Approximately 4500 clusters have been established across the country.

This study focused on natural forests with no history of plantation establishment over the past 50 years, and analyzed vegetation data from 2858 central plots (400 m^2^ each), excluding plantations. Although using only the central plot of each NFI cluster may underrepresent within-cluster heterogeneity, this approach maintains a consistent and spatially independent sampling unit across the nationwide grid and reduce within-cluster spatial dependence that could arise when multiple plots within a cluster are analyzed simultaneously. The central plots therefore provide a standardized basis for comparing stand types and their correlates at the national scale [18,19]. Among these analytical plots, 1354 were broadleaved forest plots, 659 conifer forest plots, and 845 mixed forest plots (Table S1). For each plot, species identity, diameter at breast height (DBH), and tree height were available for all tree individuals. To comprehensively evaluate plant community structure and its determinants by stand type, this study conducted analyses separately for broadleaved, conifer, and mixed stands, as well as for total stands combined. To do so, each plot was classified into one of three forest stand types based on the proportion of total basal area contributed by conifer versus broadleaved tree species (Table S2). Conifer stands were defined as plots in which conifer species accounted for ≥75% of total basal area; broadleaved stands were defined as plots in which broadleaved species accounted for ≥75% of total basal area; and mixed stands were defined as plots in which neither conifer nor broadleaved species exceeded this 75% threshold [18].

### 2.2. Phylogenetic Tree Construction and Quantification of Phylogenetic Community Structure

To analyze the phylogenetic community structure of each plot, phylogenetic trees were constructed using the *V.PhyloMaker2* package in R 4.1.2 [20], based on the tree species recorded in the 2858 plots. Four separate phylogenies were constructed for broadleaved, conifer, mixed, and total stands, comprising 203, 126, 171, and 220 woody species including trees and shrubs, respectively; these phylogenies were then used to quantify phylogenetic structure for each stand type (Figure S1). The phylogenies used here were derived from a megatree widely employed in community phylogenetic studies [20], and may therefore include uncertainty in species-level branching relationships (e.g., unresolved nodes, polytomies) and branch lengths.

As an index of community phylogenetic structure at the plot level, the standardized effect size of mean pairwise phylogenetic distance (SES.MPD) was calculated for each plot. SES.MPD is a widely used metric that quantifies the phylogenetic relatedness among species within a community. For each plot, SES.MPD was calculated as follows [3]:

$$SES.MPD=\frac{{MPD}_{obs}-{meanMDP}_{rand}}{{SD}_{rand}}$$

where MPD_obs_ is the observed mean pairwise phylogenetic distance among all possible pairs of species in a plot, and meanMPD_rand_ and SD_rand_ are the mean and standard deviation of MPD values obtained from 1000 randomly generated assemblages. The 1000 random assemblages were generated by randomly shuffling species labels across the tips of the phylogenetic tree [3,21,22,23]. This randomization procedure preserves the observed species-by-plot matrix while randomizing the phylogenetic relationships among species. Thus, species richness, species occurrence frequencies, and the spatial distribution of species are maintained in each randomization.

In general, communities with SES.MPD values less than −1.96 are interpreted as being phylogenetically clustered, whereas communities with SES.MPD values greater than 1.96 are interpreted as phylogenetically overdispersed [24,25]. SES.MPD was calculated using the *picante* package in R 4.1.2.

### 2.3. Quantification of Biotic and Abiotic Factors

To characterize biotic factors affecting phylogenetic community structure by stand type, species richness and the community-weighted means (CWMs) of specific leaf area (SLA) and maximum tree height (MH) were used. Species richness represents the amount of phylogenetic space occupied by a community and provides a baseline for interpreting phylogenetic structure [18]. CWM is an index that ecologically aggregates functional traits with large interspecific variation at the community level, thereby providing a representative value that indicates which traits are favored in community assembly [3,18].

In this study, SLA and MH were used as key traits for calculating CWMs because they are essential for plant survival and growth. SLA is a core trait that reflects resource-use strategies such as photosynthetic rate, growth rate, and nutrient turnover, whereas MH is a size-related trait that is closely associated with canopy structure, light competition ability, and long-term biomass accumulation. These traits are also closely linked to stand development: early successional communities often have higher SLA associated with fast-growing, acquisitive strategies, whereas later successional stages tend to shift toward lower SLA and more conservative resource-use strategies. Likewise, MH reflects canopy stratification and competitive dominance for light, which often become more pronounced as stand develop, leading to greater representation of tall, late-successional canopy species. Trait data for SLA and MH were obtained from a database compiled by the author’s laboratory [18]. Trait values represent species-level mean estimates and were applied consistently across plots to characterize stand-level trait composition. Because trait values are species-level means, intraspecific trait variation across local environmental gradients is not captured, which may attenuate plot-level trait–environment coupling and yield conservative trait-mediated associations. The CWM of trait *x* for each plot was calculated as:

$${CWM}_{x}=\sum_{i=1}^{n}p_{i}t_{i}$$

where CWM*_x_* is the community-weighted mean of trait *x*, *n* is the number of tree species in the plot, *t*_*i*_ is the trait value of the *i*-th species, and *p*_*i*_ is the relative basal area of the *i*-th species, calculated as the proportion of its basal area (at breast height) relative to the total basal area of all tree species in the plot [4,18].

As abiotic factors, elevation, mean annual temperature, mean annual precipitation, and stand age were used, which are key variables representing filtering effects associated with topography, climate, and forest succession. Elevation was extracted from a digital elevation model provided by the National Geographic Information Institute [18]. Mean annual temperature and mean annual precipitation were extracted from spatial climate data provided by WorldClim (https://www.worldclim.org/) [18,26]. Stand age for each plot was obtained from the NFI database as the mean number of annual rings of the five dominant trees recorded in each plot [26]. This measure primarily reflects the age of the canopy-dominant trees (i.e., canopy establishment age) and may approximate time since population establishment following past disturbance or management, but it does not uniquely distinguish time since disturbance or canopy closure.

### 2.4. Statistical Analysis

Prior to statistical analyses, all variables were log- or square-root-transformed, as appropriate, to improve linearity and normality. Subsequently, all variables were standardized to reduce differences in units and facilitate comparison of effect sizes among predictors. To minimize multicollinearity among explanatory variables, first, correlation analyses were conducted. Elevation and mean annual temperature showed a strong correlation (|r| > 0.65; Figure S2), and therefore mean annual temperature was excluded from subsequent analyses. Multicollinearity in multiple regression models was further assessed using variance inflation factors (VIFs). In all cases, VIF values were less than 3, indicating that multicollinearity was unlikely to bias the results.

To evaluate the relative importance of each predictor for phylogenetic community structure by stand type, multi-model inference was performed [27]. To avoid overfitting and obtain robust parameter estimates, model coefficients were averaged across models with a ΔAIC ≤ 2, based on Akaike’s Information Criterion (AIC) [28]. The relative importance of each variable was then quantified by summing the absolute values of its standardized regression coefficients (β) across the selected models and dividing by the total sum across all variables, yielding a proportional importance value for each predictor [23,27,28].

In addition, to elucidate the linkage structure among explanatory variables and their relationships with phylogenetic community structure, a piecewise structural equation model (pSEM) was constructed based on a conceptual model (Figure S3) [29]. The conceptual model was specified a priori based on established theory and empirical evidence linking abiotic gradients to diversity and trait composition and linking these biotic attributes to phylogenetic structure [3,18,27]. First, a full model was specified that included all plausible paths among the explanatory variables and between these variables and the phylogenetic community structure (SES.MPD). A d-separation procedure was then applied to iteratively remove non-significant paths and selected the final model as the most parsimonious model that adequately described the data [18,27]. Model fit was assessed using Fisher’s C statistic, associated *p*-values, and AIC.

All multi-model inference and pSEM analyses were conducted in R version 4.1.2 using the *MuMIn* and *piecewiseSEM* packages, respectively.

## 3. Results

**Figure 2:**
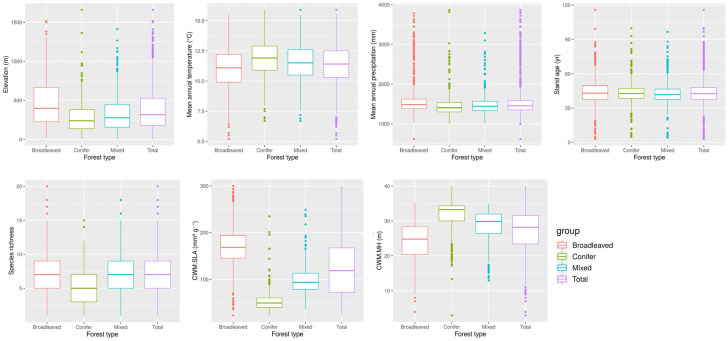
Distributions of abiotic and biotic variables across stand types in temperate forest of South Korea. Boxplots show elevation, mean annual precipitation, stand age, species richness (SR), and community-weighted means (CWM) of specific leaf area (SLA) and maximum height (MH) for broadleaved, conifer, mixed and total stands.

Across stand types, abiotic and biotic variables showed clear differences. Elevation and mean annual precipitation were higher in broadleaved stands than in conifer and mixed stands, whereas mean annual temperature showed the opposite pattern. Stand age did not differ markedly among the three stand types. Species richness (SR) was higher in broadleaved and mixed stands than in conifer stands. The community-weighted mean of specific leaf area (CWM.SLA) was highest in broadleaved stands, while the CWM of maximum tree height (CWM.MH) was higher in conifer stands than in broadleaved and mixed stands (Figure 2).

**Figure 3:**
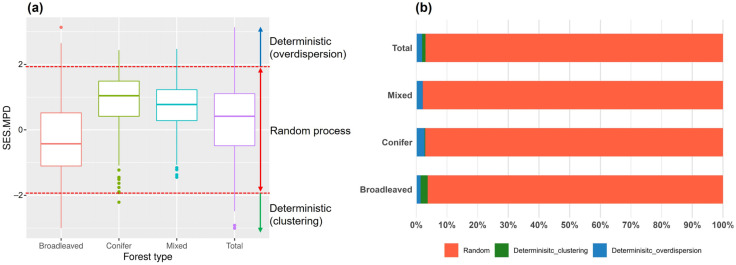
Phylogenetic community structure across stand types: (**a**) standardized effect size of mean pairwise phylogenetic distance (SES.MPD), and (**b**) classification of plots as phylogenetically clustered, overdispersed, or random based on a two-tailed α = 0.05 criterion (approximately SES.MPD ≤ –1.96 and ≥1.96).

Analysis of phylogenetic community structure showed that the mean SES.MPD value of broadleaved stands was negative (−0.28 ± 1.02), whereas conifer (0.91 ± 0.78) and mixed (0.75 ± 0.67) stands exhibited positive mean values (Figure 3). However, across the forest types, the proportion of plots classified as phylogenetically random (i.e., not significantly different from the null expectation) was much higher than the proportion showing significant phylogenetic clustering or overdispersion.

**Figure 4:**
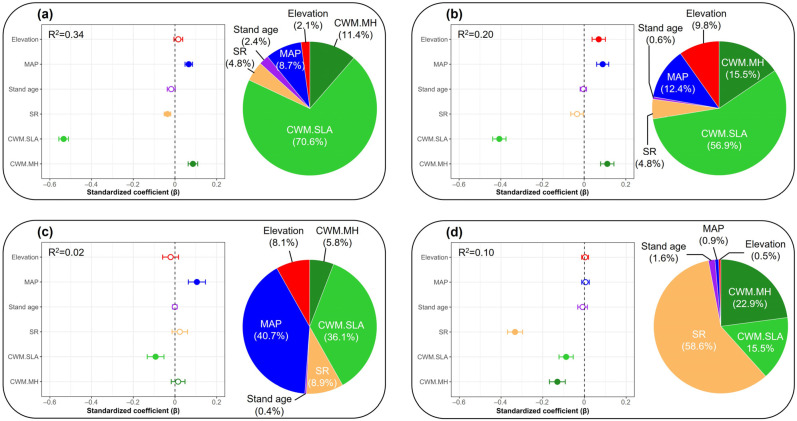
Standardized parameter estimates (circles) with standard deviations (bars) of explanatory variables on standardized effect size of mean pairwise phylogenetic distance (SES.MPD), used as a proxy of community phylogenetic structure, for (**a**) total, (**b**) broadleaved, (**c**) conifer, and (**d**) mixed stands in temperate forests of South Korea. Estimates were derived from multi-model inference tests with model averaging: (**a**) total, (**b**) broadleaved, (**c**) conifer, and (**d**) mixed forest stands. Abbreviations: MAP, mean annual precipitation; SR, species richness; CWM, community weighted mean; SLA, specific leaf area; MH, maximum height.

The multimodel inference analysis revealed that, for total stands combined (R^2^ = 0.34), CWM.SLA (β = −0.53, *p* < 0.001) had the strongest and significantly negative association on SES.MPD, and SR (β = −0.04, *p* < 0.05) also had a significant negative association. By contrast, CWM.MH (β = 0.09, *p* < 0.001) and mean annual precipitation (MAP; β = 0.07, *p* < 0.001) showed significant positive associations on SES.MPD (Figure 4a). In broadleaved stands (R^2^ = 0.20), CWM.SLA again showed the strongest negative association on SES.MPD (β = −0.41, *p* < 0.001), while CWM.MH (β = 0.11, *p* < 0.001), MAP (β = 0.09, *p* < 0.01), and elevation (β = 0.07, *p* < 0.05) all had significant positive associations (Figure 4b). In conifer stands (R^2^ = 0.02), MAP (β = 0.10, *p* < 0.05) had a significant positive association on SES.MPD, whereas CWM.SLA (β = −0.09, *p* < 0.05) had a significant negative association (Figure 4c). In mixed stands (R^2^ = 0.10), the biotic factors SR (β = −0.33, *p* < 0.001), CWM.MH (β = −0.13, *p* < 0.001), and CWM.SLA (β = −0.09, *p* < 0.05) all had significant negative associations on SES.MPD (Figure 4d).

**Figure 5:**
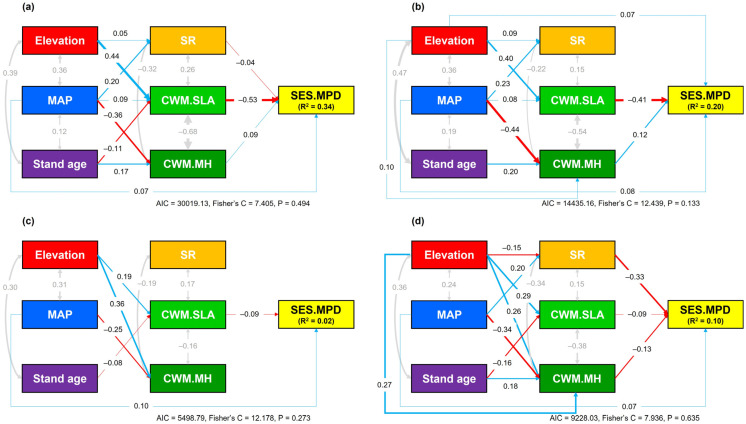
Piecewise structural equation models describing the direct and indirect pathways linking abiotic factors (elevation, mean annual precipitation, stand age) and biotic factors (species richness and trait CWMs) to the standardized effect size of mean pairwise phylogenetic distance (SES.MPD), used as a proxy for community phylogenetic structure, for (**a**) total, (**b**) broadleaved, (**c**) conifer, and (**d**) mixed stands in temperate forests of South Korea. Solid blue and red arrows indicate positive and negative effects, respectively, and gray double-headed arrows indicate covariances between variables. Standardized coefficients are shown for each path and covariance, and model fit statistics are provided. Abbreviations: AIC, Akaike information criterion; Fisher’s C, Fisher’s chi-square; SR, species richness; CWM, community weighted mean; SLA, specific leaf area; MH, maximum height.

The pSEM results showed that, for total stands combined (R^2^ = 0.34), CWM.SLA had the strongest direct negative effect on SES.MPD, while CWM.MH and SR exerted a direct positive and negative effect, respectively (Figure 5a). Abiotic factors such as elevation, MAP, and stand age affected SES.MPD indirectly through their influences on biotic variables. In particular, elevation had the largest negative indirect effect on SES.MPD (−0.23) by increasing CWM.SLA, which in turn reduced SES.MPD. In broadleaved stands (R^2^ = 0.20), CWM.SLA also had the largest direct effect on SES.MPD, and elevation showed the strongest negative indirect effect (−0.16) on SES.MPD via its positive effect on CWM.SLA (Figure 5b).

In conifer stands (R^2^ = 0.02), both MAP and CWM.SLA had direct effects on SES.MPD, although the magnitudes of these effects were relatively small (Figure 5c). Abiotic factors, namely elevation and stand age, had weak indirect effects on SES.MPD via CWM.SLA (elevation: −0.02; stand age: 0.01). In mixed stands (R^2^ = 0.10), in contrast to the other stand types, SR showed the strongest negative direct effect on SES.MPD (Figure 5d). High precipitation reduced SES.MPD indirectly (−0.07) by increasing SR, whereas higher elevation had an opposite indirect effect (0.05) on SES.MPD by reducing SR.

## 4. Discussion

The present study analyzed plot-level phylogenetic community structure of forests across South Korea and evaluated stand-type-specific correlates and linkage pathways with SES.MPD. By jointly evaluating biotic and abiotic correlates and comparing stand types, the analyses provide a national-scale, stand-type-comparative view of how phylogenetic dispersion relates to environment, functional composition, and diversity. The main findings can be summarized as follows: (1) Under the chosen null model, SES.MPD in most plots did not deviate significantly from the null expectation, indicating weak net phylogenetic structure at the national scale. (2) Mean SES.MPD differed among stand types, tending to be slightly negative in broadleaved stands and positive in conifer and mixed stands. (3) The strongest correlates of SES.MPD differed among stand types: trait composition (especially CWM.SLA) was most strongly associated with SES.MPD in total stands and broadleaved stands, MAP showed the strongest association in conifer stands, and species richness was the dominant correlate in mixed stands. (4) Abiotic gradients were linked to SES.MPD largely via indirect pathways through trait composition and species richness, highlighting trait- and richness-mediated environmental filtering. Below, these results are interpreted with careful distinction between inference, plausible explanation, and implication.

Across stand types, the high proportion of plots not significantly different from the null-model baseline suggests that net departures from null-model-relative phylogenetic dispersion are generally weak at the national scale. This does not mean that deterministic processes such as environmental filtering or competition are unimportant in Korean forests; rather, it is more reasonable to interpret the findings in this study as evidence that multiple assembly mechanisms overlap and counteract each other in space and time, leading to strong apparent randomness in the resulting patterns [30,31]. This interpretation is consistent with studies showing that combined deterministic and stochastic influences can yield weak net phylogenetic structure when evaluated across broad extents and heterogeneous conditions [1,4,30,31,32]. Importantly, the prominence of random patterns may also be amplified by averaging effects and scale mismatch across spatially heterogeneous environments and disturbance histories, and by potential phylogenetic signal dilution, all of which can reduce detectable departures from the null model baseline. Accordingly, the results are interpreted as reflecting the combined influence of random and deterministic processes, with their relative importance varying across space and stand types [28,30].

A plausible explanation for weak net departures at the national scale is the strong heterogeneity in disturbance history, management legacies, and successional pathways across Korea [18]. Opposing plot-level signals (clustering in some contexts, overdispersion in others) can partially offset one another when aggregated across the entire country, bringing SES.MPD values closer to zero overall. In addition, SES.MPD outcomes depend on methodological choices—especially the null model and the definition of the species pool. In this study, species pools were stratified by stand type because phylogenies (and associated species sets) were constructed separately for broadleaved, conifer, mixed, and total stands. Nonetheless, even within each stand type, the species pool still represents a nationwide stand-type species pool (i.e., the full set of species recorded across Korea for that stand type), which is broader than ecologically realistic pool available to many individual plots. This mismatch can reduce detectable departures from the null-model baseline and contribute to weak net phylogenetic structure at the national scale [31,33,34]. Because each stand type is standardized against its own nationwide stand-type species pool, SES.MPD comparisons among stand types are interpreted as stand-type-conditional (null-model-relative) patterns rather than as absolute effect sizes on a single common baseline [21,25,26,31].

Stand-type comparisons revealed consistent, interpretable contrasts. Broadleaved stands tended to show slightly negative mean SES.MPD, whereas conifer and mixed stands tended to show positive mean values. Broadleaved stands in South Korea are often dominated by oaks, including *Quercus mongolica*, so that species within a particular clade—especially Fagaceae—tend to repeatedly dominate. Because temperate tree floras are often dominated by a limited number of woody families and genera, repeated dominance within a few clades is not unexpected. This pattern is consistent with repeated selection under similar environmental contexts (i.e., filtering) [1,2]. Conifer and mixed stands, by contrast, often include multiple lineages (e.g., Pinus spp. together with various broadleaved taxa). Given Korea’s disturbance and management history together with ongoing natural succession, these stands likely reflect overlapping influences of niche differentiation, dispersal limitation, and historical contingencies, producing a tendency toward overdispersion in some contexts [1,33]. Mixed stands in particular can represent transitional states where conifer and broadleaved lineages co-occur, allowing multiple processes to operate simultaneously [33].

Both the multi-model inference and the pSEM consistently indicate that SES.MPD is most strongly associated with stand-type-specific biotic attributes, whereas abiotic gradients are linked to SES.MPD largely through trait- and richness-mediated pathways. Accordingly, stand-type contrasts in phylogenetic dispersion are interpreted primarily in terms of differences in functional composition and diversity, with climate and topography acting mainly as upstream constraints [31,35,36,37].

Trait-based results can be interpreted in terms of canopy and resource-use strategies while avoiding over-claiming specific mechanisms. The association between CWM.MH and SES.MPD suggests that vertical structure and competitive strategies covary with phylogenetic dispersion, consistent with studies linking canopy height to phylogenetic structure [38]. In conifer stands, relatively homogeneous composition within the stand type may partly explain weaker effect sizes and more limited variation explained by predictors. In mixed stands, the negative association between richness and SES.MPD suggests that higher richness does not necessarily translate into greater phylogenetic dispersion, potentially reflecting repeated dominance by a few major clades (e.g., pines and oaks) even as additional species accumulate—patterns that can occur during succession and stand transitions [39,40].

Stand age showed primarily indirect relationships with SES.MPD, implying that development-related changes may operate through shifts in trait composition and diversity rather than through a strong direct association. This suggests that stand age captures aspects of stand development and disturbance legacies (e.g., stand establishment following past disturbance or management), and that these temporal dynamics may first alter the functional trait composition of communities (e.g., leaf strategies, height structure), with subsequent consequences for phylogenetic community structure [37,38,39,40]. However, because Korean forests can share similar stand ages despite differing disturbance histories and management pathways, stand age alone may be an imperfect proxy for successional stage; this uncertainty could weaken or obscure stand-age-related relationships with SES.MPD and warrants cautious interpretation.

From an applied perspective, the results suggest that one-size-fits-all strategies based only on broad environmental gradients may be insufficient, and that stand-type-specific approaches are more appropriate. For broadleaved stands, management that maintains diverse leaf strategies and lineages (and avoids extreme dominance by a single clade) may better support phylogenetic and functional resilience. For conifer stands, climatic constraints—especially precipitation—should be explicitly considered alongside trait composition, given potential climate sensitivity of dominant conifer lineages. For mixed stands, maintaining diversity while managing canopy structure and avoiding extreme dominance by a few clades may help sustain compositional resilience during transitional dynamics [2,4,31,38,40,41]. Phylogenetic community structure can therefore serve as a practical indicator for planning and restoration, but interpretation must remain aligned with what SES-based metrics can and cannot support.

Finally, SES.MPD—used here to quantify phylogenetic community structure—is an indirect, null-model-relative descriptor of phylogenetic dispersion and is therefore sensitive to methodological choices. SES-based metrics can covary with species richness and species-pool definition, and estimates may also be affected by phylogenetic resolution and branch-length uncertainty (including unresolved nodes/polytomies in megatree-derived phylogenies). Moreover, linking clustering or overdispersion to specific mechanisms depends on phylogenetic signal in relevant traits and on the spatial scale of inference; at national scale, environmental heterogeneity and disturbance legacies can also produce averaging effects that weaken detectable departures from the null-model baseline. Accordingly, SES.MPD patterns are interpreted as stand-type-specific, null-model-relative deviations rather than direct evidence of particular assembly mechanisms. Trait composition was characterized using species-level mean SLA and maximum height, which does not capture intraspecific variation across plots; this may attenuate trait–environment coupling and yield conservative estimates of trait-mediated associations with SES.MPD, because local trait expression and realized height constraints can vary with microclimate, soils, and stand development.

## 5. Conclusions

This study provides a nationwide assessment of plot-level phylogenetic community structure across broadleaved, conifer, and mixed temperate forests in South Korea using the 7th National Forest Inventory. Under the chosen null model, SES.MPD in most plots did not deviate significantly from the null expectation, indicating weak net phylogenetic structure at the national scale, while mean patterns and key correlates differed among broadleaved, conifer, and mixed stands. Trait composition (especially community-weighted specific leaf area) and species richness emerged as strong stand-type-specific correlates of SES.MPD, and piecewise structural equation models suggested that abiotic gradients are linked to phylogenetic structure largely through indirect pathways via traits and richness. These results support stand-type-specific, trait- and phylogeny-informed management and restoration to enhance resilience under ongoing environmental change—for example, prioritizing trait-based species selection in broadleaved stands, accounting for climatic constraints in conifer stands, and maintaining diversity in mixed stands to promote compositional resilience. Future work should evaluate the robustness of SES-based inference by comparing multiple null-model specifications (e.g., tip-shuffling, richness- or frequency-constrained, and swap-based randomizations such as independent swap) and by using higher-resolution, DNA-based phylogenies, and it should also consider complementary phylogenetic metrics (e.g., SES.MNTD alongside SES.MPD) that capture patterns at different phylogenetic depths. In addition, future studies should incorporate additional functional traits (and, where feasible, intraspecific trait variation) and include more explicit disturbance-history and successional indicators beyond stand age to better resolve the processes underlying national-scale patterns.

## Data Availability

The data that support the findings of this study are available from Chang-Bae Lee, corresponding author, upon reasonable request.

## Supplementary Materials

The following supporting information can be downloaded at: https://www.mdpi.com/article/10.3390/biology15030268/s1, Table S1. The raw dataset used in this study. Abbreviations: MAT, mean annual temperature; MAP, mean annual precipitation; SR, species richness; CWM, community weighted mean; SLA, specific leaf area; MH, maximum height; SES.MPD, standardized effect size of mean pairwise phylogenetic distance.; Table S2. Dominant woody species and their relative abundance (%) across total, broadleaved, conifer, and mixed stands in the 7th Korean National Forest Inventory. Abundance was calculated as the proportion of the summed diameter at breast height (DBH) of each species relative to the summed DBH of all species within the corresponding stand type.; Figure S1: Phylogenetic trees constructed using V.PhyloMaker2 for (a) total, (b) broadleaved, (c) conifer, and (d) mixed stands in temperate forests of South Korea.; Figure S2: Pearson’s correlation coefficients among explanatory variables for (a) total, (b) broadleaved, (c) conifer, and (d) mixed stands in temperate forests of South Korea. Abbreviations: MAT, mean annual temperature; MAP, mean annual precipitation; SR, species richness; CWM, community weighted mean; SLA, specific leaf area; MH, maximum height.; Figure S3: Conceptual model illustrating the hypothesized effects of explanatory variables on the standardized effect size of mean pairwise phylogenetic distance (SES.MPD), used as a proxy for community phylogenetic structure in temperate forests of South Korea. The hypothesized relationships are based on evidence from previous studies. Abbreviations: MAT, mean annual temperature; MAP, mean annual precipitation; SR, species richness; CWM, community weighted mean; SLA, specific leaf area; MH, maximum height.
