# Supplementary material for: Associations of Biotic and Abiotic Factors with Phylogenetic Community Structure Across Temperate Forests in South Korea
Source: Biology (Basel). 2026 Feb 2;15(3):268. doi: 10.3390/biology15030268 (PMC12897400; doi:10.3390/biology15030268)
Supplement: Supplementary file 1 [file biology-15-00268-s001.zip › Supplementary material 2_CB Lee_01_25_2026.pdf]

Supplementary Material

Associations of biotic and abiotic factors with phylogenetic community structure across temperate forests in South Korea

Chang-Bae Lee[1,2,3]

1 Department of Forest Resources, Kookmin University, 77 Jeongneung Rd., Seongbukgu, Seoul 02707, Republic of Korea; kecolee@kookmin.ac.kr

2 Department of Climate Technology Convergence, Kookmin University, 77 Jeongneung Rd., Seongbukgu, Seoul 02707, Republic of Korea

3 Forest Carb`on Graduate School, Kookmin University, 77 Jeongneung Rd., Seongbukgu, Seoul 02707, Republic of Korea

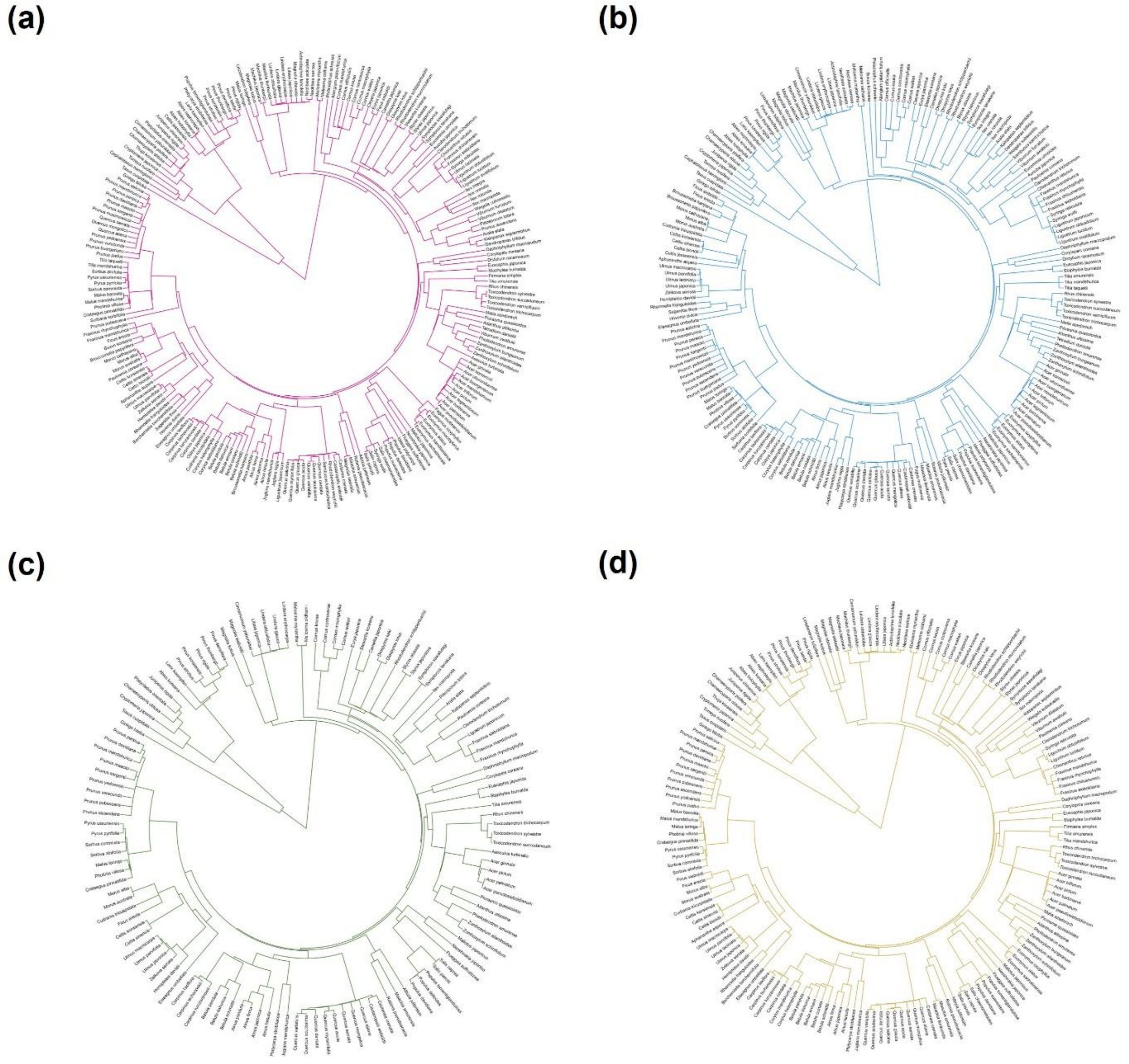

Figure S1. Phylogenetic trees constructed using V.PhyloMaker2 for (a) total, (b) broadleaved, (c) conifer, and (d) mixed stands in temperate forests of South Korea.

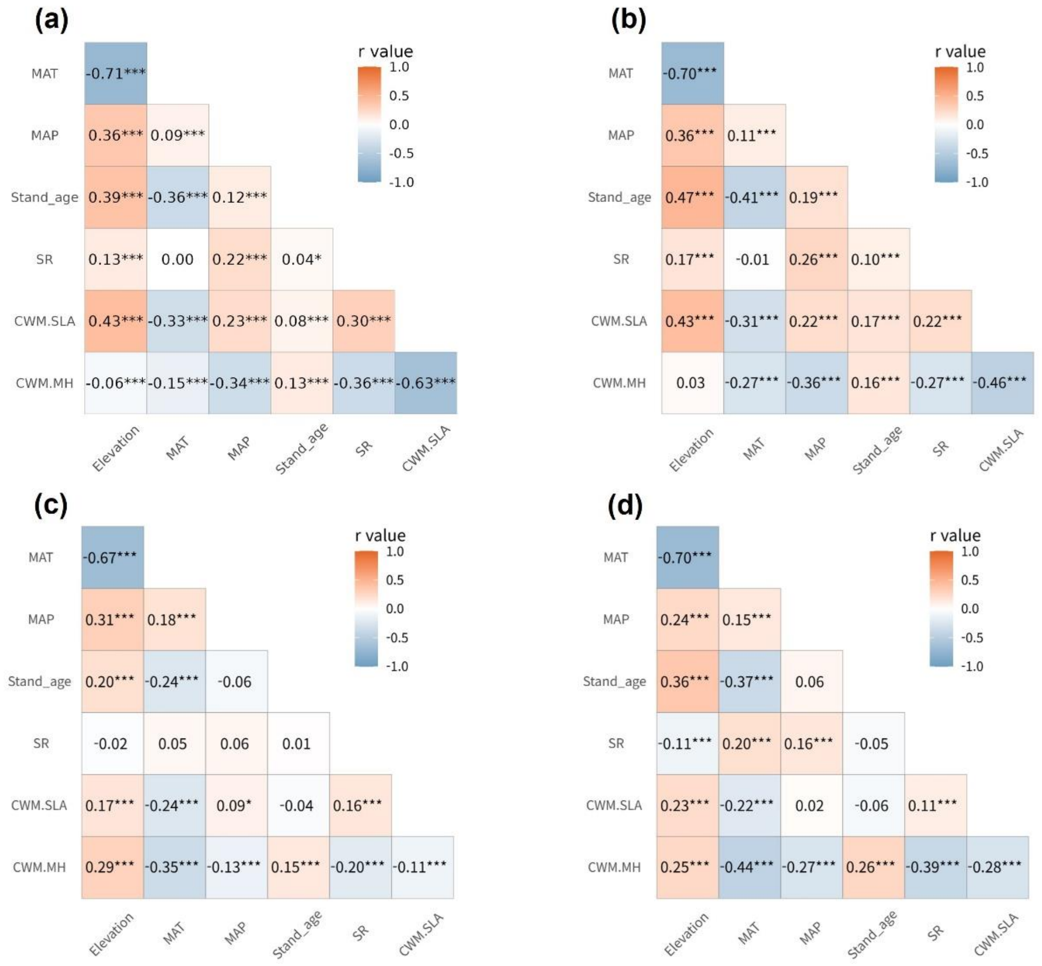

Figure S2. Pearson's correlation coefficients among explanatory variables for (a) total, (b) broadleaved, (c) conifer, and (d) mixed stands in temperate forests of South Korea. Abbreviations: MAT, mean annual temperature; MAP, mean annual precipitation; SR, species richness; CWM, community weighted mean; SLA, specific leaf area; MH, maximum height.

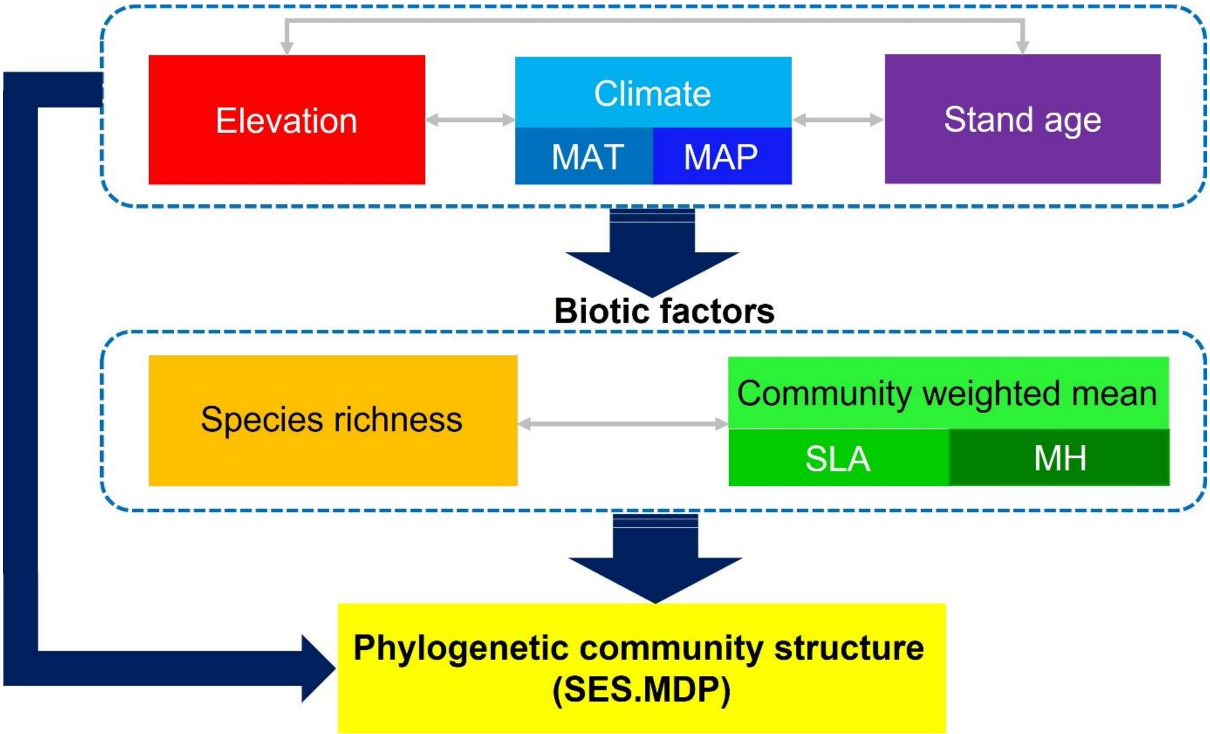

Figure S3. Conceptual model illustrating the hypothesized effects of explanatory variables on the standardized effect size of mean pairwise phylogenetic distance (SES.MPD), used as a proxy for community phylogenetic structure in temperate forests of South Korea. The hypothesized relationships are based on evidence from previous studies (Chun and Lee et al., 2018; Kim and Lee, 2021; Hu et al., 2022; Zheng et al., 2023; Lian et al., 2025). Abbreviations: MAT, mean annual temperature; MAP, mean annual precipitation; SR, species richness; CWM, community weighted mean; SLA, specific leaf area; MH, maximum height.

References

Chun, J.H.; Lee, C.B. Partitioning the Regional and Local Drivers of Phylogenetic and Functional Diversity along Temperate Elevation Gradients on an East Asian Peninsula. Sci. Rep., 2018, 8, 2853.

Hu, B.; Zhang, Y.; Yakimov, B.N.; Zhao, X.; Zhang, C. Distinguishing the Mechanisms Driving Multi-Scale Community Spatial Structure in a Temperate Forest. For. Ecol. Manag., 2022, 522, 120462.

Kim, H.; Lee, C.B. On the Relative Importance of Landscape Variables to Plant Diversity and Phylogenetic Community Structure on Uninhabited Islands, South Korea. Landsc. Ecol., 2021, 36, 209–221.

Lian, Z.; et al. Distinguishing the Mechanisms Driving Community Assembly across Growth Stages in Quercus variabilis Forests. Forests, 2025, 16, 1332.

Zheng, J.; Arif, M.; He, X.; Liu, X.; Li, C. Distinguishing the Mechanisms Driving Multifaceted Plant Diversity in Subtropical Reservoir Riparian Zones. Front. Plant Sci., 2023, 14, 1138368.

Table S2. Dominant woody species and their relative abundance (%) across total, broadleaved, conifer, and mixed stands in the 7th Korean National Forest Inventory. Abundance was calculated as the proportion of the summed diameter at breast height (DBH) of each species relative to the summed DBH of all species within the corresponding stand type.

Stand type	Species category	Scientific name	Abundance (%)
Total stands	Conifer	*Pinus densiflora*	34.1
	Broadleaved	*Quercus mongolica*	13.9
	Broadleaved	*Quercus variabilis*	10.0
	Broadleaved	*Quercus serrata*	4.8
	Conifer	*Pinus thunbergii*	3.4
	Conifer	*Pinus rigida*	3.1
	Broadleaved	*Quercus acutissima*	3.0
Broadleaved stands	Broadleaved	*Quercus mongolica*	30.4
	Broadleaved	*Quercus variabilis*	19.2
	Broadleaved	*Quercus serrata*	15.4
	Conifer	*Pinus densiflora*	7.6
	Broadleaved	*Quercus acutissima*	4.7
	Broadleaved	*Castanea crenata*	3.3
	Broadleaved	*Robinia pseudoacacia*	2.8
Conifer stands	Conifer	*Pinus densiflora*	65.8
	Conifer	*Pinus thunbergii*	6.8
	Conifer	*Pinus rigida*	6.2
	Conifer	*Pinus koraiensis*	4.8
	Conifer	*Larix kaempferi*	2.2
	Broadleaved	*Quercus variabilis*	2.1
	Broadleaved	*Cryptomeria japonica*	1.9
Mixed stands	Conifer	*Pinus densiflora*	42.5
	Broadleaved	*Quercus mongolica*	10.7
	Broadleaved	*Quercus variabilis*	10.4
	Broadleaved	*Quercus serrata*	4.4
	Conifer	*Pinus thunbergii*	4.4
	Conifer	*Pinus rigida*	4.0
	Broadleaved	*Quercus acutissima*	2.7
